# Ex Vivo Evaluation of the Sepsis Triple Therapy High-Dose Vitamin C in Combination with Vitamin B1 and Hydrocortisone in a Human Peripheral Blood Mononuclear Cells (PBMCs) Model

**DOI:** 10.3390/nu13072366

**Published:** 2021-07-10

**Authors:** Annie Lauer, Markus Burkard, Heike Niessner, Christian Leischner, Olga Renner, Claudia Vollbracht, Holger Michels, Christian Busch, Tobias Sinnberg, Sascha Venturelli

**Affiliations:** 1 Division of Dermatooncology, Department of Dermatology, University of Tübingen, 72076 Tübingen, Germany; asllauer@googlemail.com (A.L.); Heike.Niessner@med.uni-tuebingen.de (H.N.); 2 Institute of Nutritional Sciences, Nutritional Biochemistry, University of Hohenheim, 70599 Stuttgart, Germany; markus.burkard@uni-hohenheim.de (M.B.); Christian.Leischner@uni-hohenheim.de (C.L.); olga.renner@uni-hohenheim.de (O.R.); 3 Cluster of Excellence iFIT (EXC 2180) “Image Guided and Functionally Instructed Tumor Therapies”, 72076 Tübingen, Germany; 4 Pascoe Pharmazeutische Praeparate GmbH, 35394 Giessen, Germany; Claudia.Vollbracht@pascoe.de (C.V.); holger.michels@pascoe.de (H.M.); 5 Dermatologie zum Delfin, 8400 Winterthur, Switzerland; ch_busch@hotmail.com; 6 Institute of Physiology, Department of Vegetative and Clinical Physiology, University of Tübingen, 72024 Tübingen, Germany

**Keywords:** vitamin C, vitamin B1, hydrocortisone, sepsis, cytokine release

## Abstract

Sepsis is an extremely complex clinical syndrome, usually involving an excessive inflammatory response including an overshooting cytokine release that damages tissue and organs of the patient. Due to the severity of this condition, it is estimated that over 11 million people die from sepsis each year. Despite intensive research in the field, there is still no specific therapy for sepsis. Many sepsis patients show a marked deficiency of vitamin C. 9 out of 10 sepsis patients have a hypovitaminosis C, and every third patient even shows a clinical deficiency in the scurvy range. In addition, low vitamin C levels of intensive care sepsis patients correlate with a higher need for vasopressors, higher Sequential Organ Failure Assessment (SOFA) scores, and increased mortality. Based on this observation and the conducted clinical trials using vitamin C as sepsis therapy in intensive care patients, the aim of the present ex vivo study was to evaluate the effects of high-dose vitamin C alone and in a triple combination supplemented with vitamin B1 (thiamine) and hydrocortisone on the lipopolysaccharide (LPS)-induced cytokine response in peripheral blood mononuclear cells (PBMCs) from healthy human donors. We found that all corticosteroid combinations strongly reduced the cytokine response on RNA- and protein levels, while high-dose vitamin C alone significantly diminished the PBMC mediated secretion of the cytokines interleukin (IL)-10, IL-23, and monocyte chemo-attractant protein (MCP-1), which mediate the inflammatory response. However, vitamin C showed no enhancing effect on the secretion of further cytokines studied. This data provides important insights into the possible immunomodulatory function of vitamin C in an ex vivo setting of human PBMCs and the modulation of their cytokine profile in the context of sepsis. Since vitamin C is a vital micronutrient, the restoration of physiologically adequate concentrations should be integrated into routine sepsis therapy, and the therapeutic effects of supraphysiological concentrations of vitamin C in sepsis patients should be further investigated in clinical trials.

## 1. Introduction

According to the definition of 2016, sepsis is a syndrome with a “life-threatening organ dysfunction caused by a dysregulated host response to infection“. This host dysreaction to infection can lead to self-damage of tissues and organs by the immune reaction [1]. At present, sepsis is a major issue in the field of critical care medicine [2]. In clinical practice, sepsis is reflected by an increase in the Sequential Organ Failure Assessment (SOFA) score, which is used for the assessment and monitoring of sepsis patients [3,4]. Six categories are scored, each ranging from 0 to 4, and reflect an increasing severity of illness and allow some outcome prediction. Inflammatory imbalance represents the most critical basis of sepsis pathogenesis and occurs throughout the whole process of sepsis. The pathogens eliciting the response include organisms such as bacteria, fungi, parasites, and viruses [5]. Effective antimicrobial administration within the first hour of documented hypotension was associated with increased survival in adult patients with septic shock [6]. As a consequence, a timely diagnosis and therapy is essential for a good prognosis [6].

According to World Health Organization’s first global report on sepsis [7], 48.9 million cases occurred worldwide in 2017, 11 million of which with fatal outcomes. These represent 20% of all annual global deaths. Among the survivors, many remain disabled, including a high number of children [8]. In the clinics, sepsis is still a leading cause of death with mortality rates of 26.7% of hospitalized patients and 42.0% of intensive care unit (ICU) patients [9]. The pathogenesis of sepsis can be attributed to distinct pathophysiological changes like endothelial dysfunction, enhanced coagulopathy, cellular dysfunction, and cardiovascular dysfunction [10]. These symptoms can be preceded by a generalized and fulminant immune reaction following infection, involving both cellular and humoral defense mechanisms. Conditions that often cause sepsis are pneumonia, abdominal infection, kidney infection, or bloodstream infection [11]. As described in a retrospective analysis by Seymour et al., patients with sepsis can be divided into four clinical phenotypes according to host-response patterns and clinical outcomes [12]. The inappropriate host response and early activation of pro- and anti-inflammatory response makes it possible to discriminate the cytokine inflammatory profile of septic patients [12]. It can therefore be characterized by excessive inflammation, but also by a defect in the inflammatory response, e.g., an exuberance of anti-inflammatory cytokines (as in anergic states). Therapeutically, broad-spectrum antibiosis is used as soon as possible, preferably within one hour of diagnosis. Furthermore, fluid therapy is used in combination with vasoactive pharmacotherapy (e.g., norepinephrine, vasopressin, dopamine, or dobutamine) to restore hemodynamic stability [13]. When patients did not respond to the previously described procedures, the use of low-dose hydrocortisone improved the outcome of septic shock according to several systemic reviews on corticosteroid treatment [14,15,16]. Unfortunately, the benefit of hydrocortisone was not evident in all the trials conducted [16]. During the onset of sepsis, endothelial and epithelial cells as well as immune cells like lymphocytes, macrophages, and neutrophils, release pro-inflammatory mediators like tumor necrosis factor-α (TNF-α), interleukin (IL)-1, IL-6, and IL-8 followed by the production of anti-inflammatory mediators like IL-10, IL-13, or transforming growth factor-β (TGF-β) [17]. Left uncontrolled, this can lead to the development of cytokine release syndrome (CRS), which in its most severe and rapid-onset form is also known as a “cytokine storm“ and is characterized by an excessive release of proinflammatory molecules [18]. On the other hand, a surge of anti-inflammatory cytokines can increase the risk of superinfection. The role of vitamin C in the modulation of immune function has been comprehensively summarized in a recent review [19]. Prevention of infections requires dietary vitamin C intake (i.e., 100–200 mg/day) to ensure adequate cell and tissue levels. In contrast, treatment of established infections may require significantly higher (gram) doses of the vitamin to compensate for the increased inflammatory response and metabolic demand [19].

Vitamin C is one of the most potent antioxidants [20] with anti-inflammatory and immune-supportive roles [19] and it is involved as a co-factor in more than 150 metabolic functions [21]. It is particularly important for the synthesis of collagen and carnitine, the bioavailability of tetrahydrobiopterin, and thus the formation of serotonin, dopamine, and nitric oxide, the synthesis of noradrenaline, the biosynthesis of amidated peptides, the degradation of the transcription factor HIF-1α, and the hypomethylation of DNA [19]. In contrast to oral application, only the intravenous route results in pharmacological plasma levels (>220 µM) that can be reached only for a short time [22]. Critically ill patients often exhibit hypovitaminosis C (<23 μM plasma concentration), despite receiving recommended intakes via enteral and/or parenteral ICU nutritional support. In line, about 40% of septic shock patients have a vitamin C deficiency [23]. Clinical vitamin C deficiency has also been reported in patients with pneumonia [24] and recently for critical COVID-19 patients [25,26,27]. Therefore, to restore an adequate vitamin C status, the vitamin C requirement increases from 200 mg/d orally to about 6-7 g/d i.v. during the course from infection prophylaxis to the treatment of acute sepsis [28].

Additionally, in sepsis patients, vitamin B1 deficiency is estimated to range from 10 to 70% [29,30,31]. This is critical because physiologically, thiamin pyrophosphate (TPP) is a necessary cofactor in a variety of important reactions, e.g., in mitochondrial aerobic respiration or pentose pathway. It is needed for the oxidation of pyruvate to acetyl-CoA by pyruvate dehydrogenase for entering the Krebs cycle and for generating NADPH via transketolase reaction, which is important for glutathione recycling. Vitamin B1 deficiency thus can result in higher serum lactate levels due to reduced aerobic mitochondrial respiration and an impaired antioxidative cell status with higher levels of reactive oxygen species [32].

In a case series of Marik et al. the combination of hydrocortisone, vitamin C, and vitamin B1 resulted in significantly reduced mortality, as well as earlier discontinuation of vasopressors compared to patients treated conventionally [33]. To replenish low plasma vitamin C levels, i.v. administration of 1.5 g every 6 h was applied, which should result in peak plasma vitamin C concentrations of 1000 to 2000 µM [22]. The efficacy of vitamin C in the treatment of sepsis was previously confirmed in animal studies, and vitamin C was also well-tolerated in sepsis patients [34,35,36].

To further evaluate these previous results ex vivo and gain more detailed information about the effects on cytokine release, we performed experiments with peripheral blood mononuclear cells (PBMCs) and used a standardized lipopolysaccharide (LPS) stimulation (10 ng/mL) as an inducible cytokine profiling model [37]. Changes of released cytokines after treatment with different vitamin C concentrations as monotherapy (ranging from 200 to 2000 µM) or in combination with vitamin B1 (1 µM), and/or hydrocortisone (2 µM) were measured cytometrically. The vitamin B1 and hydrocortisone concentrations used within this project were based on the clinical doses used in the hydrocortisone/ascorbate/thiamine (HAT) therapy regimen and were further derived from published pharmacological studies of the compounds [38,39]. Together, the present study aimed to investigate the precise impact of vitamin C, vitamin B1, and hydrocortisone applied alone or in combination on the expression profile of sepsis-related cytokines.

## 2. Materials and Methods

### 2.1. Isolation of Human Peripheral Blood Mononuclear Cells (PBMCs)

The study population was 12 healthy human blood donors and comprised six females and six males. At the time of sample collection, the average age of females was 43.3 (23–63) and that of males was 37.5 (26–46) years. All subjects showed no symptoms of vitamin B or C deficiency. Blood drawing, sample preparation, and all following procedures were approved by the local ethics review board (approval number 208/2020 BO2). A total of 5 mL ethylenediaminetetraacetic acid (EDTA) whole blood was pipetted into a 12 mL Leukosep tube (Greiner Bio One, Frickenhausen, Germany), which was filled with 3 mL Ficoll-Paque Plus (Fisher Scientific, Schwerte, Germany). After centrifugation at 1000× *g* for 10 min, the PBMC layer was then removed and transferred to a 50 mL tube and mixed with phosphate buffered saline (PBS) (Sigma-Aldrich, Taufkirchen, Germany). After another centrifugation step at 690× *g* for 10 min, the supernatant was then aspirated, and the cell pellet resuspended in 10 mL PBS. This step was repeated twice. After centrifugation the cells were resuspended in 7 mL RPMI medium (Sigma-Aldrich). For cell counting the Countess^TM^ II FL Automated Cell Counter system (Thermo Fisher Scientific, Langenselbold, Germany) was used. To determine the cell viability the cell solution was mixed 1+1 with Trypan Blue (Sigma-Aldrich, Taufkirchen, Germany).

### 2.2. Stimulation and Treatment of PBMCs

The PBMCs were treated with vitamin C (Pascoe pharmazeutische Praeparate GmbH, Giessen, Germany), vitamin B1 (Pascoe pharmazeutische Praeparate GmbH, Giessen, Germany), and hydrocortisone (Sigma-Aldrich) either alone or in various combinations. 10 ng/mL lipopolysaccharide (LPS) from *E-coli* O111:B4 (L4391, Sigma-Aldrich, Taufkirchen, Germany) was added to the samples for 6 h to induce cytokine production. As treatment during the 6 h stimulation, vitamin B1 was used at 1 µM hydrocortisone at 2 µM and vitamin C was used in a range of 0.2 mM to 2 mM.

### 2.3. LEGENDplex^TM^ Multiplex Cytokine Analysis

For cytokine analysis from PBMC supernatant, the LEGENDplex^TM^ human inflammation panel 1 (BioLegend, Koblenz, Germany) was used. Cells were treated for 6 h, as described. Culture supernatant was used for cytokine analysis via the LEGENDplex^TM^ assay. Samples were acquired in duplicates using a BD LSRII flow cytometer (BD Biosciences, Heidelberg, Germany) and FACSDiva^TM^ Software (BD Biosciences).

### 2.4. RNA Isolation and cDNA Synthesis

After treatment, the PBMCs were harvested and total RNA was extracted from the cell pellets using the RNeasy Micro Kit (Qiagen, Hilden, Germany) according to the manufacturer’s protocol. Complementary DNA was synthesized using the Reverse-Transcriptase Kit (Thermo Fisher Scientific) with 500 ng of total RNA, 4 μL of 5× RT buffer, 0.5 μL Maxima reverse transcriptase (200 U/mL), 1 μL of random hexamer primer (100 μM), dNTP (10 mM), and RNAse-free water to a total volume of 20 μL. After pre-incubation of RNA with water for 10 min at 70 °C, the master mix was added and incubated for 10 min at 25 °C, followed by 45 min at 50 °C, and a final heat inactivation step for 5 min at 85 °C.

### 2.5. Quantitative Reverse Transcription-Polymerase Chain Reaction

Quantitative reverse transcription-polymerase chain reaction (qRT-PCR) was performed in 10 μL reaction volume with GoTaq PCR Master Mix (Promega, Walldorf, Germany) according to the manufacturer’s instructions using the Light Cycler 96 (Roche LifeScience, Penzberg, Germany). The initial denaturation step was at 95 °C for 5 min, followed by 40 cycles with 10 s each for the denaturation step at 95 °C, the annealing at 60 °C, and the elongation at 72 °C. Primer sequences are listed in Table 1.

### 2.6. Statistics

Statistical analysis was conducted with GraphPad Prism version 8.4 (GraphPad Software, San Diego, CA, USA). For multiple group comparisons, one-way ANOVA with subsequent Tukey’s multiple comparisons tests was used for *p*-value calculation and significance determination. *p*-values < 0.05 were considered statistically significant (* for *p* ≤ 0.05; **: *p* ≤ 0.01; ***: *p* ≤ 0.001; ****: *p* ≤ 0.0001).

## 3. Results

### 3.1. Testing of Different Vitamin C Concentrations during LPS Stimulation via Cytokine Release

**Figure 1:**
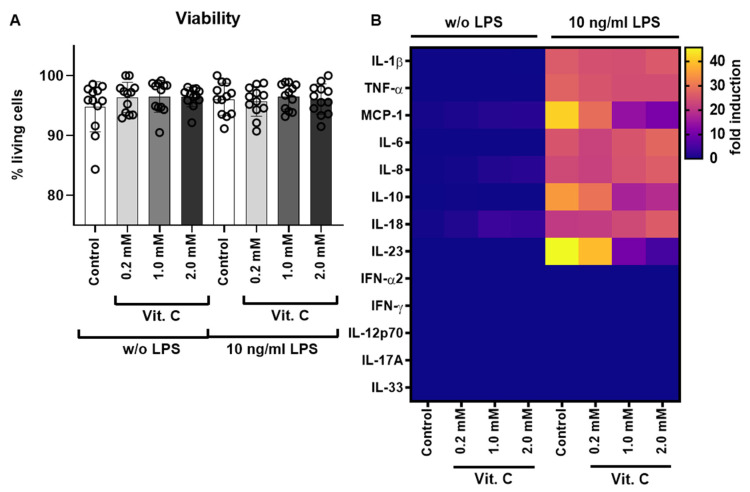
PBMC viability analysis and cytokine secretion analysis after vitamin C treatment. (**A**) The viability of the PBMCs was measured with the Countess^TM^ II FL Automated Cell Counter and a trypan blue staining. An untreated control and three different vitamin C concentrations were examined, as well as an LPS-treated control and the three vitamin C concentrations under LPS (10 ng/mL) stimulation. Shown are the mean values in percent viability and the standard deviations, with one separate data point for each donor of isolated PBMCs in all 12 donors after 6 h of treatment with different vitamin C concentrations (0.2–2 mM). (**B**) Heat map analysis of the cytokine secretion detected with the LEGENDplex^TM^ human inflammation panel 1 via flow cytometry, after stimulation of the cells with different vitamin C concentrations (0.2–2 mM) with (10 ng/mL) or without LPS; shown is the fold induction compared to the control w/o LPS. Additional data analysis stratified according to gender is available as Figure S2. IFN = interferon; IL = interleukin; LPS = bacterial lipopolysaccharides; MCP = monocyte chemo-attractant protein; PBMCs = peripheral blood mononuclear cells; TNF = tumor necrosis factor; Vit. C = vitamin C.

To test the effects of different vitamin C concentrations on LPS stimulation a total of eight treatment groups were formed for twelve different donors. The schematic workflow of the study is shown in Figure S1. Of these, six donors were female and six donors were male. PBMCs were incubated for 6 h with different concentrations of vitamin C and additionally with or without LPS to simulate a septic state. The viability of the PBMCs was measured using the Countess II^TM^ FL Automated Cell Counter. There were no differences in the viability of PBMCs before and after treatment with vitamin C and/or LPS (Figure 1A and Figure S2). Vitamin C and LPS were not cytotoxic at the concentrations used for 6 h (Figure 1A). The cell culture supernatants of the treatment groups were used to perform the LEGENDplex^TM^ assay and subsequent flow cytometry analysis. All samples were measured in biological duplicates. Data was evaluated and quantified with the help of eight standards using the software FACSDiva^TM^. The aim of the investigation of secreted cytokines was to determine whether vitamin C was able to influence the response of PBMCs to stimulation by LPS and, if so, at what concentration such an effect occurred. At the same time, this experiment investigated whether vitamin C itself had an effect on PBMCs.

**Figure 2:**
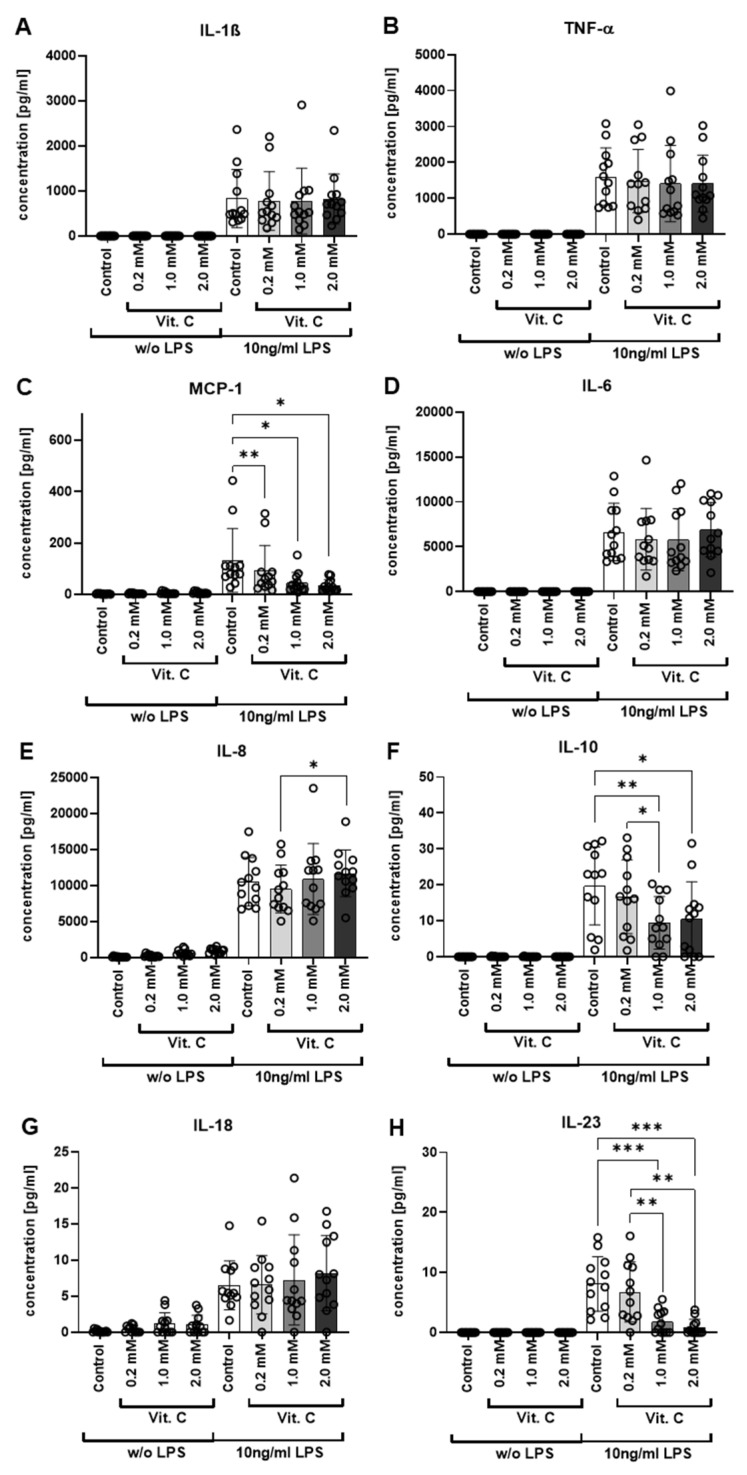
Detailed cytokine secretion analysis after vitamin C treatment. (**A**–**H**). The secreted cytokines after 6 h treatment were quantified using LEGENDplex^TM^ followed by flow cytometry. An untreated control and three different vitamin C concentrations (0.2–2 mM) were studied, as well as an LPS-treated (10 ng/mL) control and the three vitamin C concentrations under LPS stimulation. Shown are the mean values and standard deviations with one separate data point for each donor, with a measurement in duplicates for each donor. *: *p* ≤ 0.05; **: *p* ≤ 0.01; ***: *p* ≤ 0.001. Additional data analysis stratified according to gender is available as Figure S3. IL = interleukin; LPS = bacterial lipopolysaccharides; MCP = monocyte chemo-attractant protein; TNF = tumor necrosis factor; Vit. C = vitamin C.

Five of the total 13 analytes measured were below the detection limit (Figure 1B). To determine gender-specific effects, the study group was stratified by gender and analyzed accordingly. However, no gender-specific differences were found. The results of the eight detectable cytokines are shown in Figure 2A–H in detail.

The concentrations of secreted cytokines showed high standard deviations for all eight analytes above the detection limit due to the large inter-individual differences of the donors. Monocyte chemo-attractant protein (MCP-1) was hardly secreted in the treatment groups without LPS stimulation, but a significantly higher concentration of MCP-1 could be detected after LPS stimulation. Within the four treatment groups with LPS stimulation, a statistically significant decrease in MCP-1 concentration was observed when vitamin C was added compared to the controls treated with LPS alone. The decrease in MCP-1 induction upon vitamin C was concentration-dependent. Further, statistically significant changes in the secretion of cytokines occurred for IL-10. In the treatment groups without LPS stimulation, IL-10 was not secreted. However, with LPS stimulation, a significant release of IL-10 could be measured. IL-10 secretion was significantly lower in treatment groups with vitamin C at 1 (**, *p* 0.01) or 2 mM (*, *p* 0.05) compared to LPS alone.

The third molecule with significant differences between treatment groups with and without vitamin C under LPS stimulation was IL-23. In the treatment groups without LPS stimulation, IL-23 was not present in detectable amounts. In contrast, the four treatment groups with LPS stimulation showed IL-23 release. As above, a significant decrease in cytokine secretion occurred again upon vitamin C addition. At vitamin C concentrations of 1 mM and 2 mM, the reduction of IL-23 secretion compared to the control cells stimulated with LPS alone was highly significant (***, *p* 0.001). For the vitamin C concentration of 0.2 mM, no significant reduction in the concentration of IL-23 could be detected. In addition, treatment with 1 mM or 2 mM vitamin C significantly (**, *p* 0.01) reduced IL-23 secretion when compared to cells treated with 0.2 mM vitamin C.

The other five cytokines for which no significant differences were detected comparing treatment with and without vitamin C on LPS stimulation were IL-1, TNF-α, IL-6, IL-8, and IL-18 (Figure 2). The cytokines IL-1, TNF-α, and IL-6 were not secreted without LPS stimulation; the cytokines IL-8 and IL-18 were only weakly detectable without LPS stimulation. For all five, the cytokine secretion was significantly higher under LPS stimulation, but no difference between treatments with or without vitamin C could be detected. Different effects depending on the gender of the donor could not be detected either (Figure S3A–H).

### 3.2. Combination of Vitamin C with Vitamin B1 and Hydrocortisone during LPS Stimulation via Cytokine Release

**Figure 3:**
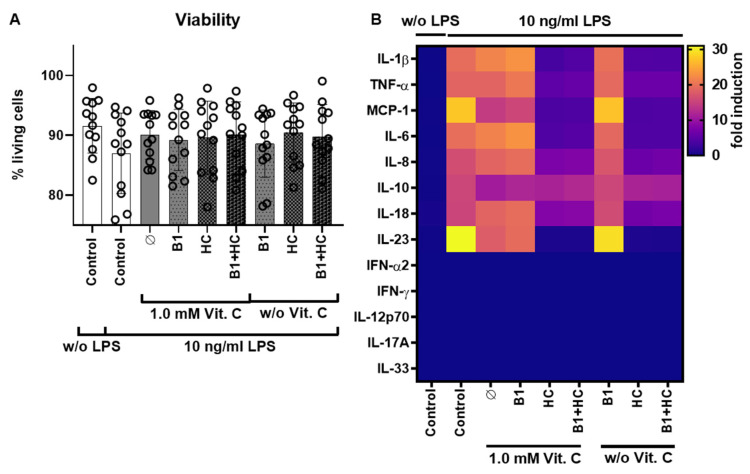
PBMC viability analysis and cytokine secretion analysis after vitamin C, vitamin B1, and hydrocortisone treatment. (**A**) The viability of PBMCs measured by Countess™ II FL Automated Cell Counter and a trypan blue staining) was at a comparable level in all donors (6 females (F), 6 males (M)) after the start of the 6 h combination treatment of Vit. C, B1, or HC alone or in combination and with (10 ng/mL) or without LPS. (**B**) Heat map analysis of the cytokine secretion measured with the LEGENDplex^TM^ human inflammation panel 1 via flow cytometry, after treatment of the cells for 6 h with either Vit. C (1 mM), B1 (1 µM), and HC (2 µM) alone or in combination and with or without LPS; shown is the fold induction compared to the control w/o LPS. Additional data analysis stratified according to gender is available as Figure S4. B1 = vitamin B1; HC = hydrocortisone; IFN = interferon; IL = interleukin; LPS = bacterial lipopolysaccharides; MCP = monocyte chemo-attractant protein; PBMCs = peripheral blood mononuclear cells; TNF = tumor necrosis factor; Vit. C = vitamin C.

In the next step, the optimized vitamin C concentration of 1 mM was combined with hydrocortisone 2 μM and vitamin B1 1 μM to simulate the clinical application of the HAT protocols. The basic experimental set-up (number of donors, freshly isolated PBMCs as human ex vivo cell model, LPS as sepsis stimulus, etc.) remained unchanged, as did the cytokine measurements via flow cytometry. As a result, the isolated PBMCs from all 12 donors (six females and six males) showed equivalent viability before (Figure S4) and after the 6 h treatment (Figure 3A), respectively.

**Figure 4:**
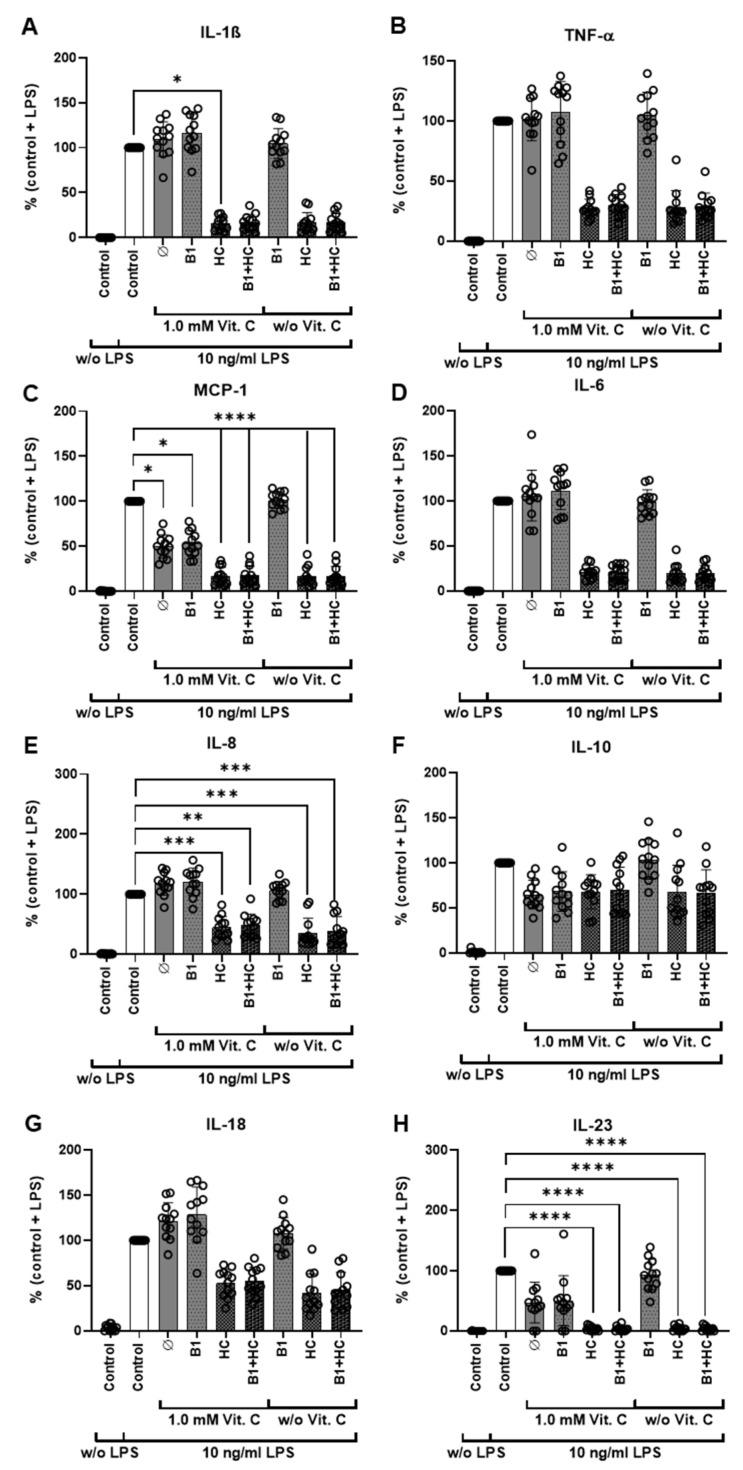
Detailed cytokine secretion analysis after vitamin C, vitamin B1, and hydrocortisone treatment. (**A**–**H**). The amount of the cytokines was determined using LEGENDplex^TM^ followed by flow cytometry after treatment of the cells for 6 h with either vitamin C, vitamin B1, or hydrocortisone alone or in combination and with or without LPS. LPS (10 ng/mL); Vit. C = Vitamin C (1 mM); B1 (1 µM); HC (2 µM); *: *p* ≤ 0.05; **: *p* ≤ 0.01; ***: *p* ≤ 0.001; ****: *p* ≤ 0.0001. Additional data analysis stratified according to gender is available as Figure S5. B1 = vitamin B1; HC = hydrocortisone; IL = interleukin; LPS = bacterial lipopolysaccharides; MCP = monocyte chemo-attractant protein; TNF = tumor necrosis factor; Vit. C = vitamin C.

Cytokine profiling was again performed by flow cytometry of the supernatants for the 13 human cytokines in all treatment groups. Consistent with the data from Figure 1 and Figure 2, the cytokines IL-10, IL-23, and MCP-1 were identified as specific therapeutic targets of vitamin C (Figure 3B and Figure 4). For better comparability among the donors, the cytokine values were normalized to the control including LPS stimulation (i.e., the absolute values in pg/mL are not shown). These controls (with LPS stimulation) were also used as a reference value for statistical evaluation.

As expected, in contrast to the results described above, treatment with 2 μM hydrocortisone alone already caused a significant reduction in cytokine release for all eight analyzed cytokines, not only for MCP-1, IL-10, and IL-23. However, the cytokine IL-10 was particularly striking, since treatment with vitamin C alone already caused a significant reduction in cytokine release, which could not be further increased by the administration of hydrocortisone. Different effects depending on gender were not detected (Figure S5).

### 3.3. Analysis of Gene Expression of PBMCs after Exposure to Vitamin C

**Figure 5:**
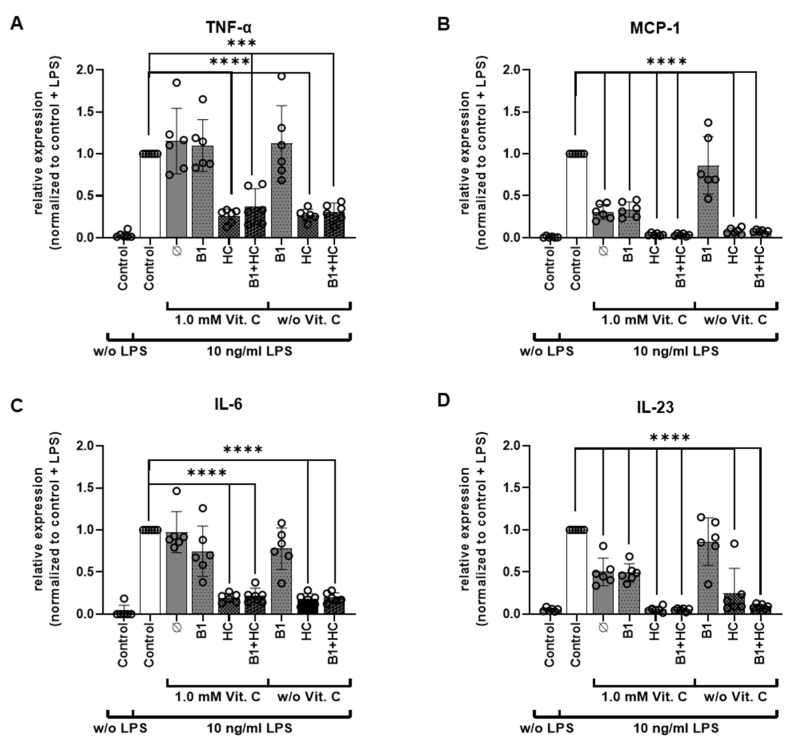
Real-time PCR analysis of TNF-α, MCP-1, IL-6, and IL-23 after vitamin C, vitamin B1, and hydrocortisone treatment. (**A**–**D**). PBMCs were treated for 6 h with either vitamin C (1 mM), vitamin B1 (1 µM), or hydrocortisone (2 µM) alone or in combination and with (10 ng/mL) or without LPS. QRT-PCR results show levels of TNF-α (**A**), MCP-1 (**B**), IL-6 (**C**), and IL-23 (**D**) mRNA expression in comparison with untreated cells and normalized to the LPS stimulated control. TBP and β-actin mRNA expression were used as housekeeping genes. ***: *p* ≤ 0.001; ****: *p* ≤ 0.0001. Additional data analysis stratified according to gender is available as Figure S6. B1 = vitamin B1; HC = hydrocortisone; IFN = interferon; IL = interleukin; LPS = bacterial lipopolysaccharides; MCP = monocyte chemo-attractant protein; PBMCs = peripheral blood mononuclear cells; qRT-PCR = quantitative reverse transcription-polymerase chain reaction; TBP = TATA box binding protein; TNF = tumor necrosis factor; Vit. C = vitamin C.

In addition, the relative expression of the four different cytokines TNF-α, IL-6, MCP-1, and IL-23 were analyzed via qRT-PCR (Figure 5A–D). In line with the LEGENDplex^TM^ assay, an increase in the expression of all analyzed cytokines was also shown after administration of LPS. In accordance with the LEGENDplex^TM^ assay, this increase was reduced by the administration of hydrocortisone or the combination of hydrocortisone and vitamin B1. The additional stimulation with vitamin C had no further reducing effect. However, a reduction of MCP-1 and IL-23 expression was mediated via vitamin C alone. Different effects depending on the gender of the donor could not be detected (Figure S6).

## 4. Discussion

Our results show that pharmacological dosages of vitamin C up to 2 mM do not influence the viability of human PBMCs from healthy donors. As postulated, vitamin C partially diminished the LPS-induced cytokine release. Among the cytokines examined, secretion of MCP-1, IL-10, and IL-23 was consistently lower with concomitant vitamin C treatment. This immunomodulatory effect of vitamin C on MCP-1, IL-10, and IL-23 is of particular interest as the septic shock is often associated with an exaggerated immune response and often fatal hypercytokinaemia [40]. Our data also confirm a dominant efficacy of the corticosteroid hydrocortisone in the combination experiments with vitamin B1 and vitamin C. In this context, it is remarkable that the effect of vitamin C on IL-10 secretion equals the effect of hydrocortisone. However, the combination of vitamin C and hydrocortisone showed no additional effect in terms of reduced cytokine release for all cytokines tested when compared with hydrocortisone alone. Interestingly, vitamin B1 had no influence on cytokine release.

IL-10 is an anti-inflammatory cytokine. During infection, it inhibits the activity of T helper cells (Th1) cells, natural killer cells, and macrophages, all of which are required for optimal pathogen clearance, but at the same time contribute to tissue damage. As a result, IL-10 can both impede pathogen clearance and enhance immunopathology. IL-10 thus has a bifunctional regulatory capacity in sepsis, and it cannot be generalized whether downregulation by, e.g., vitamin C or hydrocortisone is beneficial or harmful per se. Many different cell types produce IL-10, and the major source of IL-10 varies. The anti-inflammatory action of IL-10 is clearly underlined by its negative correlation with TNF-α and IL-6. IL-10 functions as a temporal regulator of the transition from early reversible sepsis to the late phase of irreversible shock. The onset of lethality in IL-10^−/−^ mice occurred significantly earlier than in IL-10^+/+^ mice and was associated with 15-fold-higher serum levels of TNF-α and IL-6. [41]. IL-10 pretreatment could prevent the TNF response to LPS in mice [42]. In a human study, the IL-10/lymphocyte ratio was significantly higher in non-surviving patients than in surviving patients, indicating sepsis-induced IL-10-related immunosuppression [43].

The role of IL-23 in sepsis pathology is becoming more and more evident. Increased activity of the IL-17/IL-23 pathway exhibits detrimental effects on sepsis-induced lung inflammation. Inhibitors of IL-23 are therefore speculated to have beneficial effects on the course of sepsis [44,45].

There is emerging evidence of a strong correlation of high levels of MCP-1 with organ dysfunction and mortality in sepsis patients. Moreover, corresponding animal models indicate a prominent role for MCP-1 in sepsis [46,47,48,49,50,51]. Consequently, attempts were made to target MCP-1 in systemic inflammation. Thus, prophylactic and therapeutic treatment with bindarite, a blocker of MCP-1 synthesis, significantly protected mice from sepsis and endotoxemia, as evidenced by attenuation of neutrophil activity in the lungs and liver [52].

Therefore, knowledge of the exact influence of each therapeutic component on the cytokine profile is of central importance in the evaluation of new therapeutic approaches for sepsis, such as the combination of vitamin C, vitamin B1, and hydrocortisone.

Our finding that vitamin B1 neither influenced cytokine release nor modulated the effects mediated by vitamin C or hydrocortisone is in line with a randomized double-blind placebo-controlled trial using thiamine as a metabolic resuscitator in septic shock. In this study, thiamine failed to improve lactate levels or other outcomes in the overall group of patients with septic shock and elevated lactate. However, patients with thiamine deficiency possibly had a benefit [53]. Although thiamine seems to provide some renal protection in septic shock [54], Miyamoto et al. showed in an observational study that there is no association between thiamin administration and the 28-day mortality in patients with septic shock in Japan [55].

Vitamin C appears to be able to both prevent and treat respiratory and systemic infections by enhancing various immune cell functions [19,56]. Traditionally, these are correlated to antioxidant properties (<10 mM plasma concentration) and have been repeatedly confirmed in vitro. So far, different trials have been published that were designed to prove the Marik protocol [33]. Fowler et al. published the CITRIS-ALI trial: In this preliminary study of patients with sepsis and ARDS, a 96-h infusion of vitamin C compared with placebo did not significantly improve organ dysfunction scores or alter markers of inflammation and vascular injury [57]. Interestingly, the overall mortality probability was lowered significantly, especially in the first 96 h, but unfortunately was not taken from the study coordinators as the primary outcome and was, therefore, underpowered [58]. A second trial, the so-called VITAMINS trial, compared the treatment with intravenous vitamin C, hydrocortisone, and thiamine with intravenous hydrocortisone alone in patients with septic shock [59]. The combination therapy did not significantly improve overall survival or free of vasopressor administration over seven days. These findings suggest that treatment with intravenous vitamin C, hydrocortisone, and thiamine does not lead to a more rapid resolution of a septic shock compared with intravenous hydrocortisone alone [60]. However, the patients receiving the vitamin C containing combination therapy showed lower SOFA scores on day three. Similarly, the A TESS trial could not show improvements in organ function upon vitamin C and thiamine administration in the early phase of septic shock when compared with placebo [61,62,63]. Also, the ACTS trial, in which patients with septic shock were treated with a combination of vitamin C, corticosteroids, and vitamin B1 and compared to placebo, did not result in a significant reduction in SOFA scores during the first 72 h after enrollment, but the patients showed more “shock-free“ days [64]. Meanwhile, different meta-analyses have been conducted on vitamin C in sepsis and critically ill patients. The results are controversial; while some see a reduction in mortality, a shorter hospital stay and/or a reduced need for vasopressors, these effects are not confirmed in other analyses [65,66]. Of note is a meta-analysis that sees effects depending on the chosen vitamin C dose [63].

The management of septic patients is a serious challenge due to syndrome complexity and heterogeneity. Since vitamin B1 did not affect LPS-induced cytokine secretion, a rationale can be established to modify Marik’s protocol [33], eliminating vitamin B1 and replacing it, for example, with vitamin D. Severe vitamin D deficiency was described to be independently associated with increased mortality in adult patients with sepsis [67,68]. Therefore, the determination of procalcitonin and vitamin D were recommended for prediction of septic prognosis in patients [68]. An adjuvant vitamin D therapy improved a sepsis score and diminished the incidence of septic shock in neonates and children [69,70].

Another important issue is hypotension in sepsis patients. Although the work of Juraschek et al. suggests a moderate blood pressure-lowering effect of vitamin C supplementation [71], which could worsen hypotonia in patients, this was not seen in the clinical trials in which vitamin C was used in an amount of about 1.5 g per infusion every six hours. Rather, there was either no effect on the administration of vasopressors or even a reduction in the time in which vasopressor administration was necessary [72].

Since other trials, partly very large ones, are registered at clinicaltrial.gov and are recruiting, it remains to be seen what these studies will show. The current clinical trial data do not show general significant benefits for patients with septic shock receiving therapy with vitamin C, corticosteroids, and vitamin B1. However, possible unidentified subgroups of patients might benefit from the combination as suggested by Marik et al. [33], the timing seems to be a decisive matter (time from diagnosis to therapy should be <6 h) and also dosage is an important factor as studies showed higher effectiveness of higher dosages (200 mg/kg/day) [36]. The latter finding was recently confirmed by a meta-analysis of nine randomized control trials with a total of 584 patients (301 in the intervention group and 283 in the control group), which demonstrated a reduction of ICU mortality (OR = 0.60 95% CI [0.42, 0.85], *p* = 0.004) and dosage of vasopressors applied (SMD = 20.88 95% CI [21.48, 20.29], *p* = 0.003) in the intervention group treated with vitamin C (compared to placebo). However, the ICU length of stay did not differ between the two groups. Therefore, multicenter and randomized controlled trials are needed to further clarify the safety and effectiveness of using vitamin C to treat patients with sepsis and septic shock [73].

Our ex vivo study provides further evidence for the immunomodulatory effects of vitamin C on human PBMCs in the context of LPS-induced cytokine response. Nevertheless, there are a few limitations involved in the study which should be addressed in future research. The general effect of vitamin C on IL-10, IL-23, and MCP-1 was evident, however, the effects were not determined in samples from septic patients. It would be valuable to extend the research with samples from septic patients as well as a larger number of probands and to complement this with micro-nutrient analysis. In addition, the current investigation was carried out with isolated PBMCs, and it might be important to confirm these findings in whole blood samples as an ex vivo model and next, in an appropriate animal model, also to investigate the effects on survival. Further, LPS represents a stimulus from gram-negative bacteria, but sepsis can also be caused by other pathogens including gram-positive bacteria. Therefore, similar effects should be confirmed using additional pathogen-derived components like lipoteichoic acid from gram-positive bacteria. All these aspects are worthy of further investigation.

## 5. Conclusions

Vitamin C had a modulatory capacity on certain inflammatory cytokines. Further in-depth analysis including more sophisticated sepsis models are needed to evaluate the impact of vitamin C on the attenuation of a cytokine storm and the sepsis therapy. Despite the interesting results of vitamin C in this ex vivo PBMC sepsis model, the effect of hydrocortisone on cytokine release is the most relevant. Given that vitamin B1 had no influence on cytokine release, Marik’s protocol could be modified by replacing vitamin B1 for example with vitamin D.

As vitamin C is a vital micronutrient, restoring physiologically adequate concentrations should be a routine part of any therapeutic approach. Whether supraphysiological concentrations of vitamin C are associated with additional therapeutic benefits in sepsis patients needs to be verified in further clinical trials.

## Figures and Tables

**Table 1:** Primer sequences (Forward (fo) and reverse (re) primer sequences for quantitative real-time PCR).

Name	Sequence
IL6_fo	5′-cacagacagccactcacctc
IL6_rev	5′-ttttctgccagtgcctcttt
TNFa_fo	5′-ctcttctgcctgctgcactttg
TNFa_rev	5′-atgggctacaggcttgtcactc
MCP1_fo	5′-agaatcaccagcagcaagtgtcc
MCP1_rev	5′-tcctgaacccacttctgcttgg
IL23A_fo	5′-gagccttctctgctccctgata
IL23A_rev	5′-gactgaggcttggaatctgctg
TBP_fo	5′-tgcacaggagccaagagtgaa
TBP_rev	5′-cacatcacagctccccacca
ActinB_fo	5′-ttgttacaggaagtcccttgcc
ActinB_rev	5′-atgctatcacctcccctgtgtg

## Supplementary Materials

The following are available online at https://www.mdpi.com/article/10.3390/nu13072366/s1, Figure S1: Schematic workflow of the study; Figure S2: PBMC viability analysis before treatment with vitamin C; Figure S3: Detailed cytokine secretion analysis of male and female PBMC samples after vitamin C treatment; Figure S4: PBMC viability analysis before treatment with vitamin C, vitamin B1 and hydrocortisone; Figure S5: Detailed cytokine secretion analysis of male and female PBMC samples after vitamin C, vitamin B1 and hydrocortisone treatment; Figure S6: Real-time PCR analysis of TNF-α, MCP-1, IL-6, and IL-23 after vitamin C, vitamin B1, and hydrocortisone treatment of PBMCs from male and female donors
