# Supplementary material for: Ex Vivo Evaluation of the Sepsis Triple Therapy High-Dose Vitamin C in Combination with Vitamin B1 and Hydrocortisone in a Human Peripheral Blood Mononuclear Cells (PBMCs) Model
Source: Nutrients. 2021 Jul 10;13(7):2366. doi: 10.3390/nu13072366 (PMC8308809; doi:10.3390/nu13072366)
Supplement: Supplementary file 1 [file nutrients-13-02366-s001.zip › nutrients-1264624-SI.pdf]

“*Ex vivo* evaluation of the sepsis triple therapy high-dose vitamin C in combination with vitamin B1 and hydrocortisone in a human peripheral blood mononuclear cells (PBMCs) model”

Supplementary figures

Lauer et al.

Figure S1

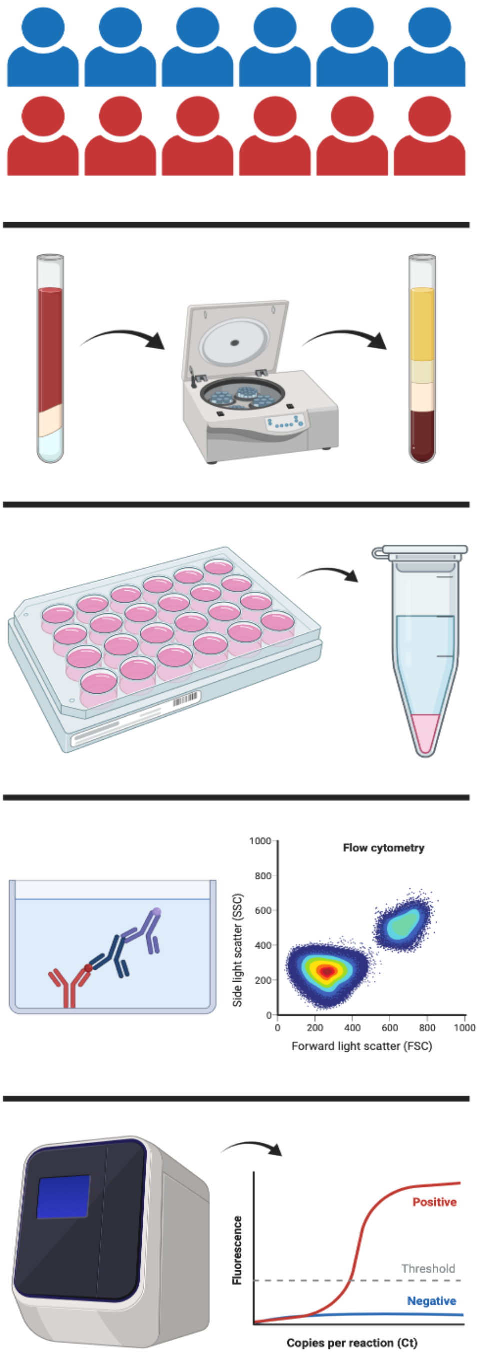

Figure S1. **Schematic workflow of the study**. Blood collection from 12 healthy donors (6 women, 6 men) and isolation of PBMCs. Simulation of an infectious event by using LPS as a stimulus. Incubation of PBMCs with different drug combinations for 6 h, followed by recovery of the PBMCs (pellet) and the supernatant. Analysis of the secreted cytokines in the supernatant by immunoassay and subsequent flow cytometry. Investigation of gene expression in the PBMCs by qRT-PCR. LPS = bacterial lipopolysaccharides; PBMCs = peripheral blood mononuclear cells; quantitative reverse transcription-polymerase chain reaction (qRT-PCR).

Figure S2

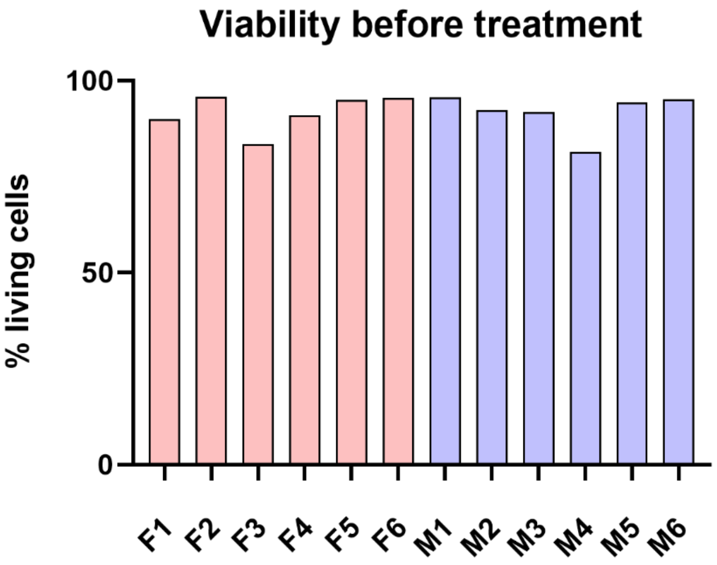

Figure S2. **PBMC viability analysis before treatment with vitamin C.** The viability of the PBMCs was measured automatically with the Countess II FL Automated Cell Counter and a trypan blue staining. The values are shown as a percentage for the individual donors, with one measurement for each donor (F = female donors; M = male donors). PBMCs = peripheral blood mononuclear cells.

Figure S3

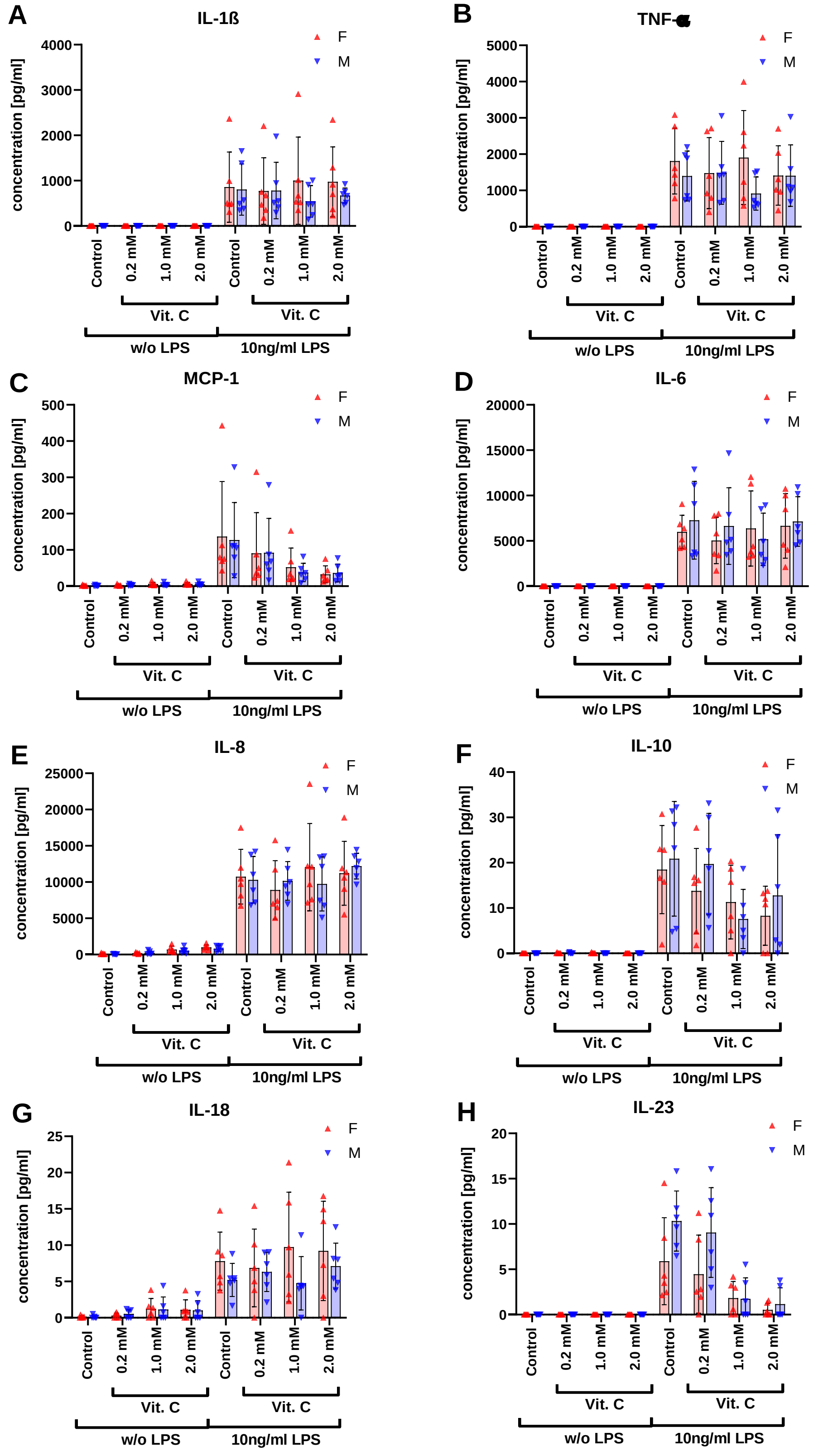

Figure S3. Detailed cytokine secretion analysis of male and female PBMC samples after vitamin C treatment. A-H. The amount of the cytokines was determined after 6 h treatment using LEGENDplex followed by flow cytometry. An untreated control and three different vitamin C concentrations were examined, as well as an LPS-treated control and the three vitamin C concentrations (0.2 – 2 mM) under LPS (10 ng/ml) stimulation. The male and female donors are shown separately. Shown are the mean values and standard deviations with one separate data point for each donor, with a measurement of two biological replicates for each donor (F = female donors; M = male donors). IL = interleukin; LPS = bacterial lipopolysaccharides; MCP = monocyte chemo-attractant protein; PBMCs = peripheral blood mononuclear cells; TNF = tumor necrosis factor; Vit. C = vitamin C.

Figure S4

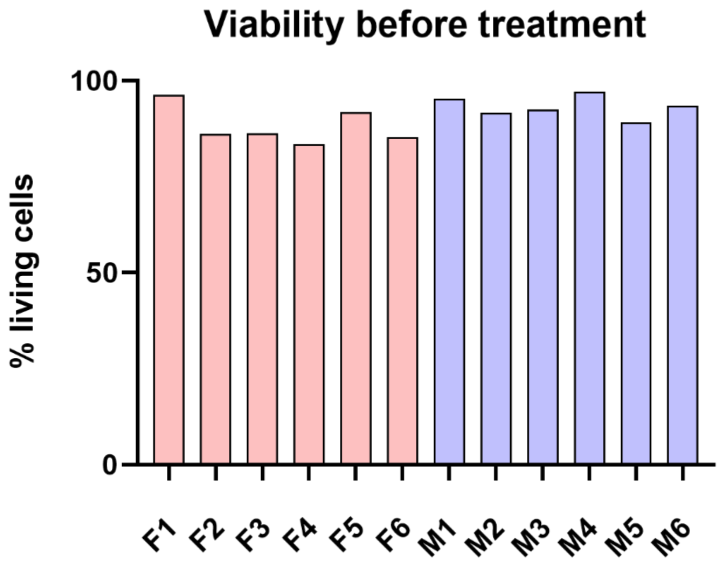

Figure S4. **PBMC viability analysis before treatment with vitamin C, vitamin B1 and hydrocortisone.** The viability of the PBMCs was measured automatically with the Countess II FL Automated Cell Counter and a trypan blue staining. The values are shown as a percentage for the individual donors, with one measurement for each donor (F = female donors, M = male donors). PBMCs = peripheral blood mononuclear cells.

Figure S5

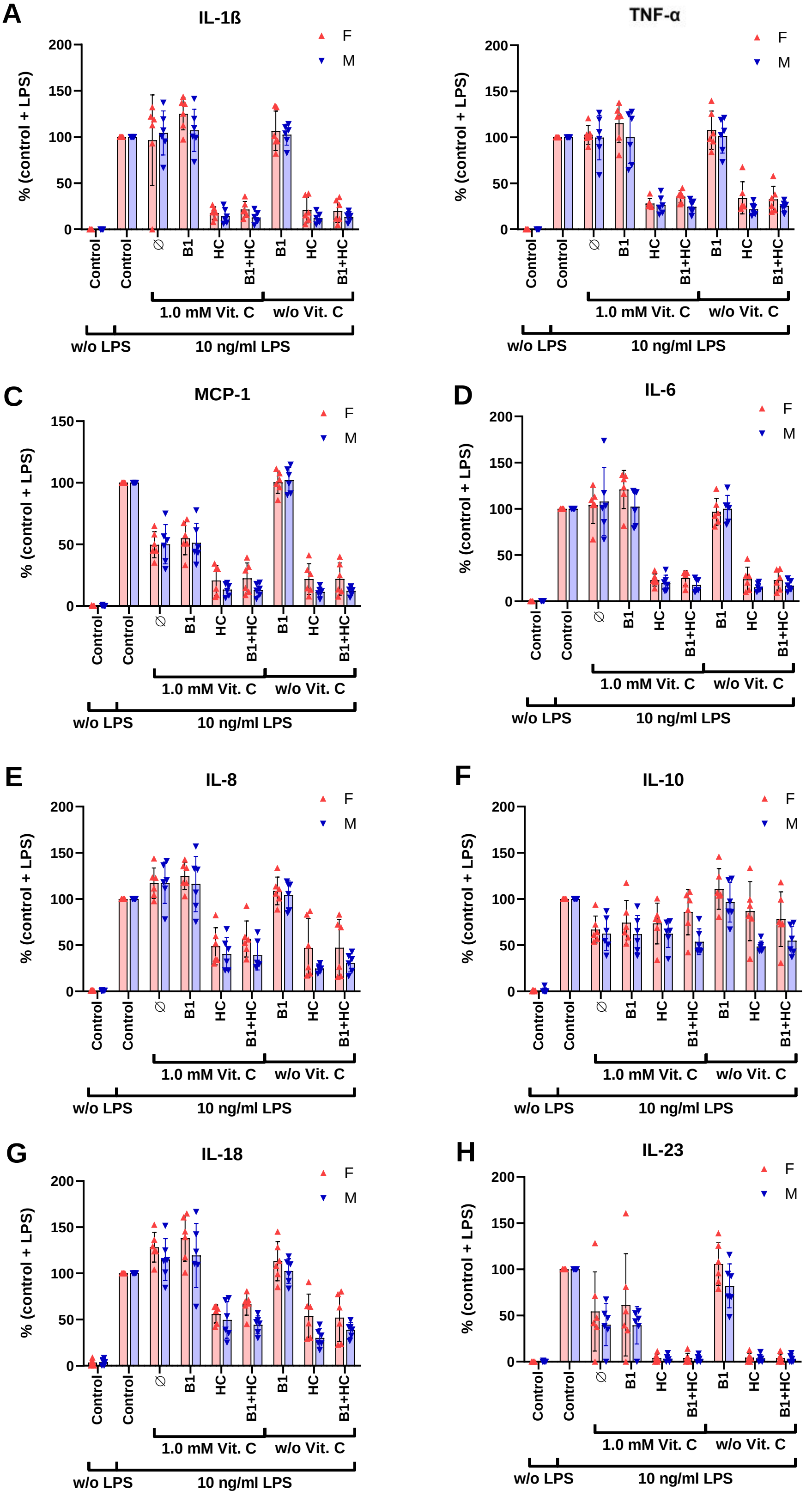

Figure S5. **Detailed cytokine secretion analysis of male and female PBMC samples after vitamin C, vitamin B1, and hydrocortisone treatment. A-H.** The amount of the cytokines was determined using LEGENDplex followed by flow cytometry after treatment of the cells for 6 h with either vitamin C (1 mM), vitamin B1 (1 μM), and hydrocortisone (2 μM) alone or in combination and with (10 ng/ml) or without LPS. The male (M) and female (F) donors are shown separately. B1 = vitamin B1; HC = hydrocortisone; IL = interleukin; LPS = bacterial lipopolysaccharides; MCP = monocyte chemo-attractant protein; PBMCs = peripheral blood mononuclear cells; TNF = tumor necrosis factor; Vit. C = vitamin C.

Figure S6

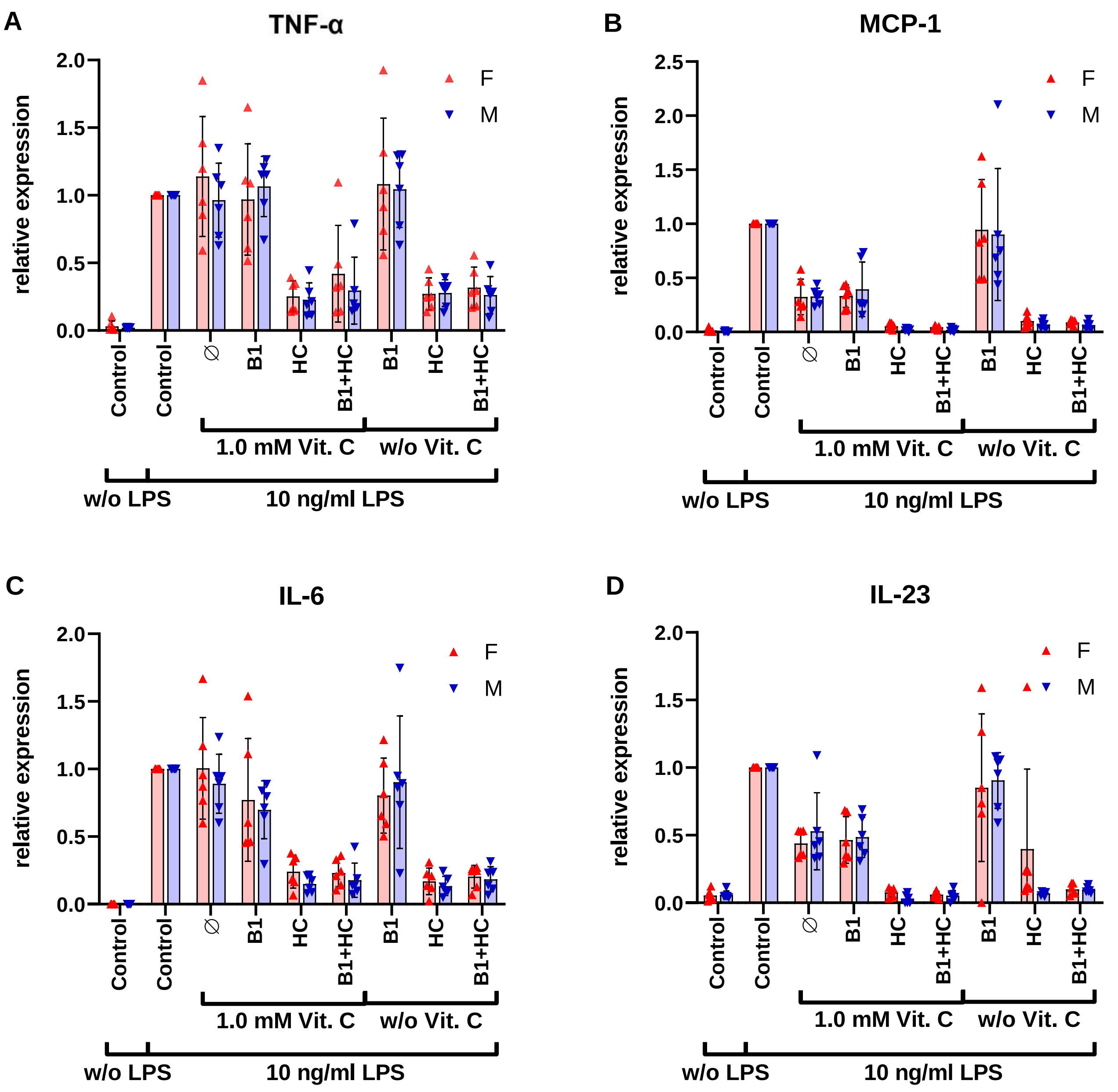

Figure S6. Real-time PCR analysis of TNF-α, MCP-1, IL-6, and IL-23 after vitamin C, vitamin B1, and hydrocortisone treatment of PBMCs from male and female donors. A-D. PBMCs were treated for 6 h with either vitamin C (1 mM), vitamin B1 (1 μM), and hydrocortisone (2 μM) alone or in combination and with (10 ng/ml) or without LPS. QRT-PCR results show levels of TNF-α (A); MCP-1 (B), IL-6 (C), and IL-23 (D) mRNA expression in comparison with untreated cells and normalized to the LPS stimulated control. TBP and β-actin mRNA expression were used as housekeeping genes. The male (M) and female (F) donors are shown separately. B1 = vitamin B1; HC = hydrocortisone; IFN = interferon; IL = interleukin; LPS = bacterial lipopolysaccharides; MCP = monocyte chemo-attractant protein; PBMCs = peripheral blood mononuclear cells; qRT-PCR = quantitative reverse transcription-polymerase chain reaction; TBP = TATA box binding protein; TNF = tumor necrosis factor; Vit. C = vitamin C.
